# Supplementary material for: Empirical analysis of the text structure of original research articles in medical journals
Source: PLoS One. 2020 Oct 8;15(10):e0240288. doi: 10.1371/journal.pone.0240288 (PMC7544105; doi:10.1371/journal.pone.0240288)
Supplement: S1 Table — Means and standard deviations for the number of paragraphs per section, the total number of paragraphs (Total), the number of tables, figures and references by journal and year of publication. The last column provides absolute frequencies and relative frequencies (in parenthesis) for the availability of supplementary material (Suppl). BMJ: The BMJ, JAMA: The Journal of the American Medical Association, NEJM: The New England Journal of Medicine, PLOS: PLOS Medicine. (DOCX) [file pone.0240288.s005.docx]

| **Journal** | **Year** | **Introduction** | **Methods** | **Results** | **Discussion** | **Total** | **Tables** | **Figures** | **References** | **Suppl** |
| --- | --- | --- | --- | --- | --- | --- | --- | --- | --- | --- |
| BMJ | 2005 | 2.93 ± 1.17 | 7.40 ± 3.38 | 7.97 ± 4.70 | 7.97 ± 2.34 | 26.27 ± 4.51 | 3.70 ± 1.82 | 1.30 ± 1.15 | 26.60 ± 10.65 | 14 (0.47) |
|  | 2010 | 3.20 ± 1.06 | 11.17 ± 5.42 | 10.30 ± 5.98 | 10.73 ± 4.34 | 35.40 ± 9.73 | 3.37 ± 2.08 | 3.27 ± 2.60 | 42.20 ± 21.08 | 16 (0.53) |
|  | 2015 | 3.80 ± 0.81 | 11.80 ± 4.27 | 8.70 ± 3.86 | 10.10 ± 5.09 | 34.40 ± 9.45 | 3.07 ± 1.66 | 2.53 ± 2.10 | 47.07 ± 31.36 | 26 (0.87) |
| JAMA | 2005 | 3.40 ± 1.28 | 10.20 ± 3.71 | 8.73 ± 2.90 | 9.97 ± 2.62 | 32.30 ± 6.48 | 3.97 ± 1.59 | 1.63 ± 1.35 | 36.10 ± 16.33 | 0 (0.00) |
|  | 2010 | 3.00 ± 0.91 | 11.80 ± 2.95 | 7.97 ± 3.22 | 9.07 ± 2.63 | 31.83 ± 5.09 | 3.67 ± 1.21 | 1.67 ± 1.37 | 33.70 ± 9.54 | 19 (0.63) |
|  | 2015 | 3.10 ± 0.88 | 11.30 ± 3.70 | 10.47 ± 3.60 | 8.93 ± 2.39 | 33.80 ± 7.14 | 3.03 ± 1.19 | 2.17 ± 1.34 | 38.50 ± 16.10 | 29 (0.97) |
| Lancet | 2005 | 2.93 ± 1.11 | 9.97 ± 3.23 | 7.47 ± 4.54 | 8.97 ± 3.36 | 29.33 ± 7.75 | 3.13 ± 1.68 | 1.97 ± 1.38 | 30.07 ± 9.07 | 10 (0.33) |
|  | 2010 | 3.10 ± 1.16 | 11.00 ± 4.13 | 9.53 ± 3.89 | 9.13 ± 3.59 | 32.77 ± 9.09 | 3.37 ± 1.99 | 3.50 ± 1.76 | 36.77 ± 15.56 | 18 (0.60) |
|  | 2015 | 3.00 ± 1.02 | 14.07 ± 5.28 | 8.97 ± 3.36 | 9.90 ± 5.26 | 35.93 ± 11.02 | 2.80 ± 1.10 | 3.50 ± 1.72 | 33.97 ± 14.43 | 27 (0.90) |
| NEJM | 2005 | 2.97 ± 1.10 | 9.60 ± 3.35 | 8.83 ± 2.23 | 6.93 ± 1.23 | 28.33 ± 4.89 | 2.93 ± 1.20 | 2.33 ± 1.30 | 29.10 ± 8.14 | 12 (0.40) |
|  | 2010 | 2.80 ± 0.96 | 9.90 ± 2.87 | 9.00 ± 3.28 | 6.47 ± 1.83 | 28.17 ± 5.55 | 2.60 ± 1.10 | 2.27 ± 1.26 | 34.00 ± 8.43 | 29 (0.97) |
|  | 2015 | 3.07 ± 1.05 | 9.43 ± 3.28 | 9.57 ± 2.91 | 7.17 ± 1.76 | 29.23 ± 5.32 | 2.50 ± 1.07 | 2.27 ± 1.01 | 28.00 ± 8.77 | 30 (1.00) |
| PLOS | 2005 | 3.93 ± 1.26 | 9.27 ± 4.50 | 9.33 ± 5.30 | 8.47 ± 4.42 | 31.00 ± 11.42 | 2.53 ± 2.49 | 3.63 ± 2.67 | 41.70 ± 15.54 | 15 (0.50) |
|  | 2010 | 3.93 ± 1.80 | 10.53 ± 4.93 | 8.57 ± 3.94 | 10.20 ± 3.37 | 33.23 ± 7.78 | 3.37 ± 1.92 | 3.90 ± 4.40 | 52.23 ± 48.74 | 26 (0.87) |
|  | 2015 | 4.60 ± 2.16 | 14.20 ± 6.69 | 11.57 ± 5.90 | 10.40 ± 4.12 | 40.77 ± 11.53 | 3.93 ± 1.96 | 4.47 ± 3.27 | 53.43 ± 18.60 | 30 (1.00) |
