# Supplementary material for: Empirical analysis of the text structure of original research articles in medical journals
Source: PLoS One. 2020 Oct 8;15(10):e0240288. doi: 10.1371/journal.pone.0240288 (PMC7544105; doi:10.1371/journal.pone.0240288)
Supplement: S1 Appendix — (DOCX) [file pone.0240288.s006.docx]

**Appendix**

The null hypothesis is that PLOS has the same number of paragraphs as the other four journals. Formally, we look at this hypothesis as a contrast hypothesis . The linear contrast is , which is the unweighted average over the journals *B* BMJ, *J* JAMA, *L* Lancet and *N* the NEJM without *P* PLOS Medicine because we assume identical variances between journals and because the sample size is identical. The asymptotic distribution of the contrast is with the contrast vector being . Effects were estimated from a linear mixed model without intercept, journal as fixed effect and year as random effect.
